# Space and time on the membrane: modelling Type VI secretion system dynamics as a state-dependent random walk

**DOI:** 10.1098/rsos.230284

**Published:** 2023-11-01

**Authors:** Jonathan Miller, Philip J. Murray

**Affiliations:** Department of Mathematics, University of Dundee, Dundee, UK

**Keywords:** type six secretion system, spatio-temporal modelling, random walk

## Abstract

The type six secretion system (T6SS) is a transmembrane protein complex that mediates bacterial cell killing. The T6SS comprises three main components (transmembrane, baseplate and sheath/tube complexes) that are sequentially assembled in order to enable an attacking cell to transport payloads into neighbouring cells. A T6SS attack disrupts the function of essential cellular components of target cells, typically resulting in their death. While the assembled T6SS adopts a fixed position in the cell membrane of the attacking cell, the location of the firing site varies between firing events. In *Serratia marcescens*, a post-translational regulatory network regulates the assembly and firing kinetics of the T6SS in a manner that affects the attacking cell’s ability to kill target cells. Moreover, when the ability of membrane complexes to reorient is reduced, an attacking cell’s competitiveness is also reduced. In this study, we will develop a mathematical model that describes both the spatial motion and assembly/disassembly of a firing T6SS. The model represents the motion of a T6SS on the cell membrane as a state-dependent random walk. Using the model, we will explore how both spatial and temporal effects can combine to give rise to different firing phenotypes. Using parameters inferred from the available literature, we show that variation in estimated diffusion coefficients is sufficient to give rise to either spatially local or global firers.

## 1. Introduction

Bacterial cells, which typically range from 0.5 to 5.0 μm in length, are classified by morphological features such as shape (coccus or bacillus) and whether they are Gram-negative or -positive. Cellular sub-components include the cell wall, cytoplasmic membrane, periplasm and cytoplasm [1].

*Serratia marsescens* is a rod-shaped, Gram-negative facultative anaerobe and opportunistic pathogen of the family Yersiniaceae; *S. marsescens* is commonly involved in hospital-acquired infections, particularly catheter-associated bacteremia, urinary tract and wound infections [2].

Secretion systems play an important role in bacterial virulence [3,4]. Eleven different classes of secretion systems have been identified (namely the general (Sec), twin-arginine translocation (Tat) and Type I–IX secretion systems) [5–7]. Secretion systems can enable attacking cells to translocate payloads into target cells and cause cellular damage, cell death and tissue damage as well as hindering immune system response [5]. In this study, we will consider the Type VI secretion system (T6SS).

**Figure 1.**
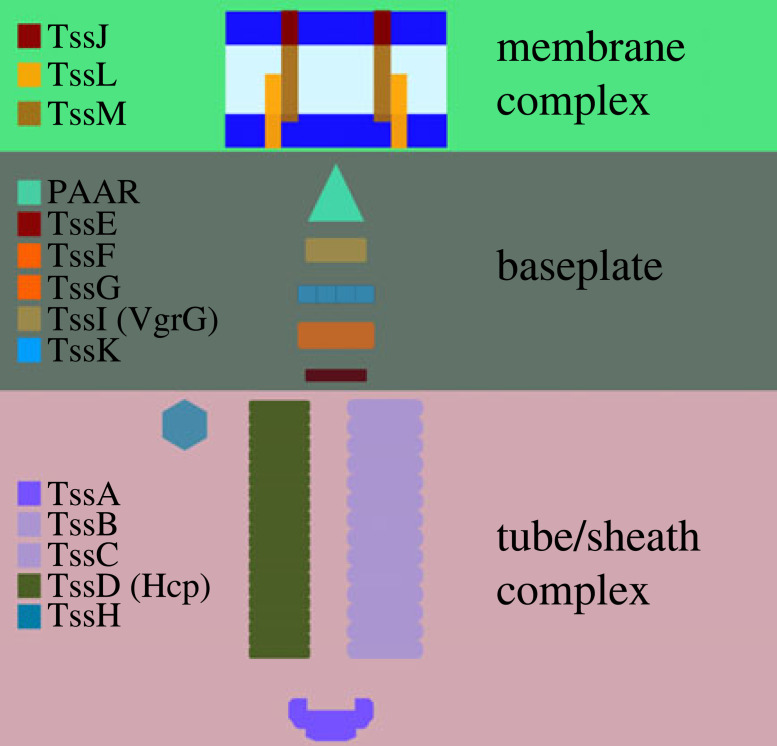
A schematic illustration of a T6SS. Each complex is defined by the proteins which make up the MC, baseplate and tube/sheath.

The T6SS is a large transmembrane complex that comprises three distinct yet connected structures: the membrane complex (MC); the baseplate; and the sheath/tube complex (figure 1). The T6SS assembles in a sequential out-to-in manner: after the MC nucleates at the outer membrane [8], the baseplate attaches to the cytoplasm-facing portion of the MC, forming a basal complex (BC) that anchors the T6SS to the cellular membrane [9]. The T6SS sheath/tube, a tubular structure responsible for the contraction and firing of the T6SS, assembles from the BC and is built inwards towards the interior of the cell. Upon firing of an assembled T6SS, the sheath contracts and the payload, which carries effector proteins that can disable the function of target cells [9], is projected out of the attacking cell.

Fluorescence imaging of labelled molecular components (figure 1) has allowed for the direct characterization of T6SS assembly in individual cells. The appearance of distinct, fluorescent foci (e.g. TssB/TssM/TssL) can be used to identify the sequential states of T6SS assembly and disassembly. For example, double-fluorescent imaging indicates that although each tube/sheath complex is associated with a corresponding membrane complex, the converse is not true; in *S. marcescens* the ratio of TssB (baseplate) to TssL/TssB (BC) double foci is 1 : 2.7 [10]. Moreover, real-time imaging of fluorescent foci indicates that the T6SS can assemble and fire in repeated cycles on a time scale of minutes [10,11] and that firing locations are distributed across the cell membrane [8].

Upon formation, the tube/sheath complex (TssB) and inner components of the membrane complex (TssM and TssL) are approximately fixed in position in the cell membrane [10]. However, clusters of TssJ, in *S. marcescens* at least, are observed to be motile on a time scale of seconds [10], suggesting that the membrane complex can spatially reorient on the membrane. When T6SSs are perturbed such that they do not reorient their firing direction, their competitive edge is diminished compared to wild-type [10–13].

Perturbation of a number of different kinases (e.g. PpkA) and phosphatases (e.g. PppA) has been shown to affect T6SS formation and, ultimately, the ability of T6SS-hosting cells to compete [11–13]. In the absence of PppA, the orientation of the T6SS remained fixed, though repetitive firings still occurred. During this time, the competitiveness of the ΔPppA strains was reduced [11]. Thus, the kinetics of various aspects of T6SS assembly and disassembly are thought to directly affect the firing cell’s effectiveness.

Previous multicellular models of T6SS competition have investigated how T6SS-mediated competition affects the population dynamics of competing cells [14,15]. The modelling has identified, for example, that when colonies of target cells are sufficiently large they can evade a T6SS attack and ultimately grow exponentially [16]. Moreover, simulations have highlighted that recently killed target cells can act as a physical barrier that protects neighbouring cells from attack [15]. In these studies, the spatio-temporal dynamics of the T6SS are modelled statistically (i.e. the position and time of the next T6SS fire are sampled from prescribed statistical distributions).

In this study we will develop a mathematical model that describes the reorientation and firing of T6SS in a single cell. Using the model we will investigate how consideration of both space and time can give rise to spatially local or global T6SS firing. The layout is as follows: in §2 we present methods; in §3 we explore numerical simulations of the model; and, finally, in §4 we conclude with a discussion.

## 2. Methods

### 2.1. Variables

**Figure 2.**
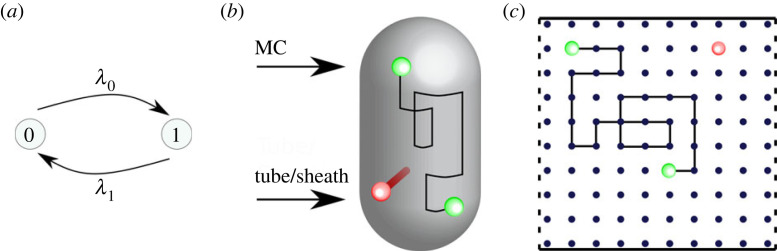
A schematic illustration of the model. (*a*) A T6SS can switch between states at rates *λ*_0_ and *λ*_1_. (*b*) A rendered capsule with both modelled states of the T6SS. State 0 (green) and State 1 (red). (*c*) In State 0, the T6SS undergoes a random walk on a regular lattice. In State 1 the position of the T6SS is fixed. Dashed lines—periodic boundary conditions. Solid lines—hard wall boundary conditions.

The surface of the cell membrane is represented by a two-dimensional domain, Ω. The position of the MC is represented by spatial coordinates **x**(*t*), where *t* represents time. The state of each MC is represented by a binary variable, *s*(*t*), such that in State 0 (*s*(*t*) = 0) the MC position can reorient on the cell membrane. In State 1 (*s*(*t*) = 1) the MC position is fixed (figure 2).

#### 2.1.1. Dynamics

To capture the sequential assembly of a T6SS, the dynamics of the state variable are approximated as being a random telegraph signal (i.e. a Markov jump process that is continuous in time and discrete in state space). In State 0 *s*(*t*) can switch to the firing state, State 1, at a rate *λ*_0_. In State 1 *s*(*t*) can switch to State 0 at a rate *λ*_1_ (figure 2*a*).

To model reorientation, it is assumed that the membrane complex undergoes a state-dependent random walk on the cell membrane: in State 0 the MC moves with diffusion coefficient, *D*_0_, while in State 1 the position of the T6SS is fixed, i.e.

2.1 $$D(s(t))=\left\{ \begin{array}{cc} D_{0},\; & s(t)=0,\;\\ 0,\; & s(t)=1.\; \end{array}\right.$$

This model could capture the observed random motion of TssJ [11]. Alternatively, it could phenomenologically describe the complete dissociation of a T6SS and the transport of molecular components to another location via an independent mechanism. It is noted that in the limit of a sufficiently large diffusion coefficient, it can approximate spatially homogeneous reorientation.

### 2.2. Implementation

The geometry of the rod-shaped *S. marcescens* cell is approximated as being cylindrical (figure 2*b*). The cell surface, Ω, is approximated as being a *L* × *W* rectangular domain that is discretized into squares of size Δ*x* × Δ*x*. One pair of boundaries is periodic (cylindrical) while the other has a hard boundary (no flux condition on hemisphere ends; see figure 2*c*). The time interval [0, *T*] is discretized into time steps of length Δ*t*.

The random walk is represented by a space-jump process such that the MC hops to a neighbouring lattice site at rate

2.2 $$h(s(t))=\left\{ \begin{array}{cc} h_{0},\; & s(t)=0,\;\\ 0,\; & s(t)=1.\; \end{array}\right.$$

The probability of hopping in a given time step is thus given by *h*(*s*(*t*))Δ*t*. Note that it is assumed that

2.3 $$h_{0}=\frac{D_{0}}{\Delta x^{2}}.$$

The probability of reactions occurring in time Δ*t* are *λ*_0_Δ*t* and *λ*_1_Δ*t*.

### 2.3. Statistical approach

The waiting times in States 0 and 1 are exponentially distributed, i.e.

2.4 $$T_{0}∼\mathrm{Exp}(\lambda_{0})\;\mathrm{and}\;T_{1}∼\mathrm{Exp}(\lambda_{1}).$$

Thus, the time for a T6SS to undergo a cycle (transition from State 0 to State 1 and back to State 0) is

$$T_{\mathrm{oc}}=T_{0}+T_{1}.$$

The expected waiting times are given by

$${\bar{T}}_{0}=\frac{\ln2}{\lambda_{0}}\;\mathrm{and}\;{\bar{T}}_{1}=\frac{\ln2}{\lambda_{1}},$$

respectively, and the expected time for one cycle is

2.5 $${\bar{T}}_{\mathrm{oc}}={\bar{T}}_{0}+{\bar{T}}_{1}.$$

The distance travelled by the T6SS in a time $\tilde{T}$, *d*, is computed using a taxicab metric. Representing the MC in a Euclidean coordinate system, **x(t)** = [*x*(*t*), *y*(*t*)], we define

$$\begin{array}{rl} \delta_{x}(\tilde{T}) & =|x(\tilde{T})-x(0)|,\\ d_{x}(\tilde{T}) & =\left\{ \begin{array}{cc} L-\delta_{x},\; & \delta_{x}>\frac{L}{2},\;\\ \delta_{x},\; & \mathrm{otherwise},\; \end{array}\right.\\ \mathrm{and}\;\;\;\;\;\;\;d_{y}(\tilde{T}) & =|y(\tilde{T})-y(0)|. \end{array}$$

The total distance is therefore

2.6 $$d(\tilde{T})=d_{x}(\tilde{T})+d_{y}(\tilde{T}).$$

Letting $P(d_{\tilde{T}})$ represent the probability distribution function of observing an MC moving a distance $d_{\tilde{T}}$ after time $\tilde{T}$, the expected distance is given by

$${\bar{d}}_{\tilde{T}}=\int_{0}^{\infty }P(d_{\tilde{T}})\;d_{\tilde{T}}dd_{\tilde{T}}.$$

Below we will consider a fixed simulation time ($\tilde{T}=T$) and the time taken for one cycle ($\tilde{T}=T_{\mathrm{oc}}$). In the latter case, the simulation time is a stochastic variable.

### 2.4. Parametrization

Parameters were estimated primarily using observations made in *S. marcescens*. To estimate the transition rates, it is noted that the ratio of TssL to TssB foci in *S. marcescens* is observed to be 2.7 : 1 [10]. Moreover, TssB foci are co-localized with TssL foci but the converse is not true. These data are indicative of a staged T6SS formation process whereby MCs are initially formed on the membrane and can act as an initiation site for tube/sheath formation. Assuming statistical independence of foci, we interpret observations of foci distributed over large numbers of cells at a single time point in the context of the proposed model by assuming that

$$\frac{T_{0}}{T_{1}}=\frac{\lambda_{1}}{\lambda_{0}}=2.7.$$

As State 0 is observed to last of the order of minutes [10], it is assumed that *λ*_0_ = 1/60 s^−1^. To estimate the hopping rate for the T6SS MC, we consider measured diffusion coefficients, *D*_0_, of different components of the cell membrane (table 2) and define the corresponding hopping rate using equation (2.3).

**Table 2.** A table of parameter values.

parameter	description	value	unit	source
*W*	width	3.2	μm	[28]
*L*	length	2.0	μm	[28]
*D*_0_	diffusion coefficient	0.0049–2.5	μm^2^ s^−1^	see table 1
*λ*_0_	transition rate	0.017	s^−1^	[10,11]
*λ*_1_	transition rate	0.045	s^−1^	[10,11]

#### 2.4.1. Estimated mean displacement

In an infinite domain, the mean distance travelled by **x**(*t*) is estimated by adapting the classical result for the mean-squared displacement of a diffusing particle (table 1) to account for state-dependent diffusion. It is assumed that the expected displacement after time, *T*, is given by

**Table 1.** Measured membrane diffusion coefficients in different bacterial species.

species	value (μm^2^ s^−1^)	description	source
*Pseudomonas aeruginosa*	0.2	periplasm	[17]
*Escherichia coli*	2.6	periplasm	[18]
*Escherichia coli*	0.0049–0.22	plasma membrane	[18–24]
*Salmonella typhimurium*	0.02	outer membrane	[25]
*Escherichia coli*	0.006–0.15	outer membrane	[26,27]

2.7 $$d=2\sqrt{\frac{\lambda_{1}}{\lambda_{0}+\lambda_{1}}D_{0}T}.$$

Substitution for the expected time for one cycle (see equation (2.5)) yields an estimate for the expected distance travelled between successive firing events given by

2.8 $$d\approx 2\sqrt{\frac{\lambda_{1}}{\lambda_{0}+\lambda_{1}}D_{0}\ln2\left(\frac{1}{\lambda_{0}}+\frac{1}{\lambda_{1}}\right)}=2\sqrt{\ln2\frac{D_{0}}{\lambda_{0}}}.$$

Given parameter values in table 2 we expect *d* ∈ [0.9, 20.4] μm.

## 3. Results

To explore a role for spatio-temporal dynamics in the regulation of T6SS firing, a model was formulated in which a T6SS MC can both move on the cell membrane and stochastically fire (see figure 2 and §2). The state of a T6SS MC is represented by the binary variable, *s*(*t*). In State 0 (*s*(*t*) = 0) the MC is motile while in State 1 (*s*(*t*) = 1) it is bound to the cell membrane. It is assumed that the MC stochastically switches between states at rates *λ*_0_ and *λ*_1_ (see §2 and figure 2*a*). The cell membrane is represented by a spatial domain, Ω, and the position of the MC is represented by coordinate x(t)∈Ω. When the MC is in the unbound state (*s*(*t*) = 0), it is assumed that it undergoes a random walk. When the BC is formed (*s*(*t*) = 1), its position is assumed to be fixed on the cell membrane (figure 2*b*,*c*). The firing of the T6SS is represented by a switch from State 1 to State 0.

The capsule-like geometry of a *S. marcescens* cell membrane is approximated by a planar spatial domain with periodic boundary conditions (figure 2*c*). The spatial domain is discretized using a regular lattice and the random walk is implemented as a space-jump process. For simplicity, a single T6SS is considered. The model is parametrized using observations of T6SS behaviour in *S. marcescens* (see §2.4). Transition rates are estimated using observations of T6SS kinetics obtained using live imaging (see §2.4).

**Figure 3.**
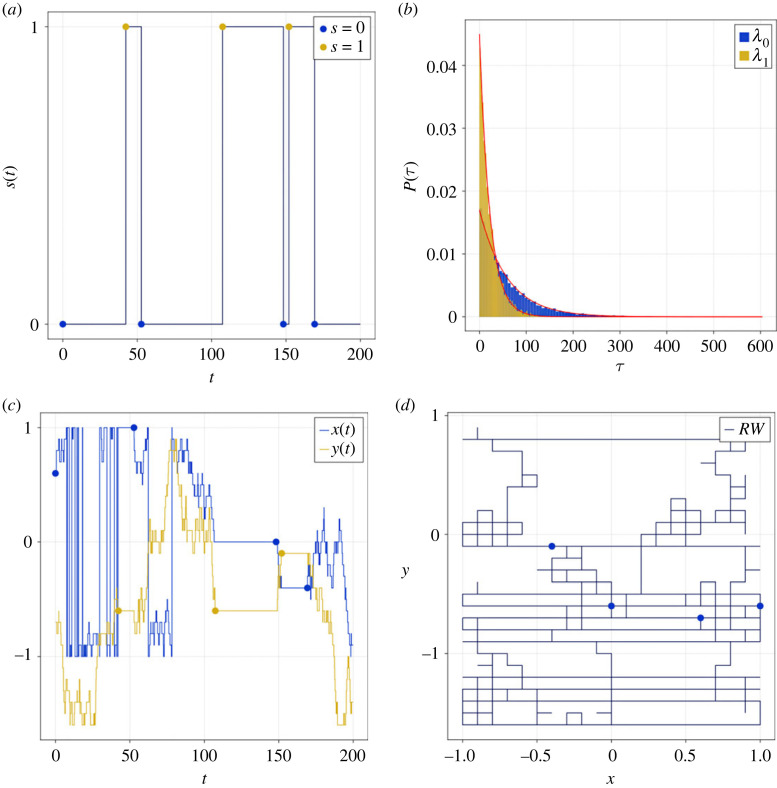
An example of model dynamics. (*a*) A realization of the state variable, *s*(*t*), is plotted against time. (*b*) The waiting time distributions for States 0 and 1 are distributed exponentially. Red lines represent theoretical results given by equation (2.4). (*c,d*) T6SS coordinates are represented as time series (*c*) and in the *xy* plane (*d*). Markers represent transitions between States 0 and 1. See table 2 for parameter values.

Numerical solutions of the model demonstrate that, as expected, the state variable *s*(*t*) behaves like a stochastic telegraph process (see figure 3*a* for a representative realization). The MC spends some time in State 0 before switching to State 1. T6SS firing and disassembly is represented by a switch from State 1 back to State 0. The waiting times in each of the states are, as expected, exponentially distributed (figure 3*b*). When the baseplate is in State 0, the MC undergoes a random walk while in State 1 its position is fixed (figure 3*c*,*d*). Hence the effective hopping rate of the baseplate is a function of both the base-line hopping rate and the transition rates between states.

**Figure 4.**
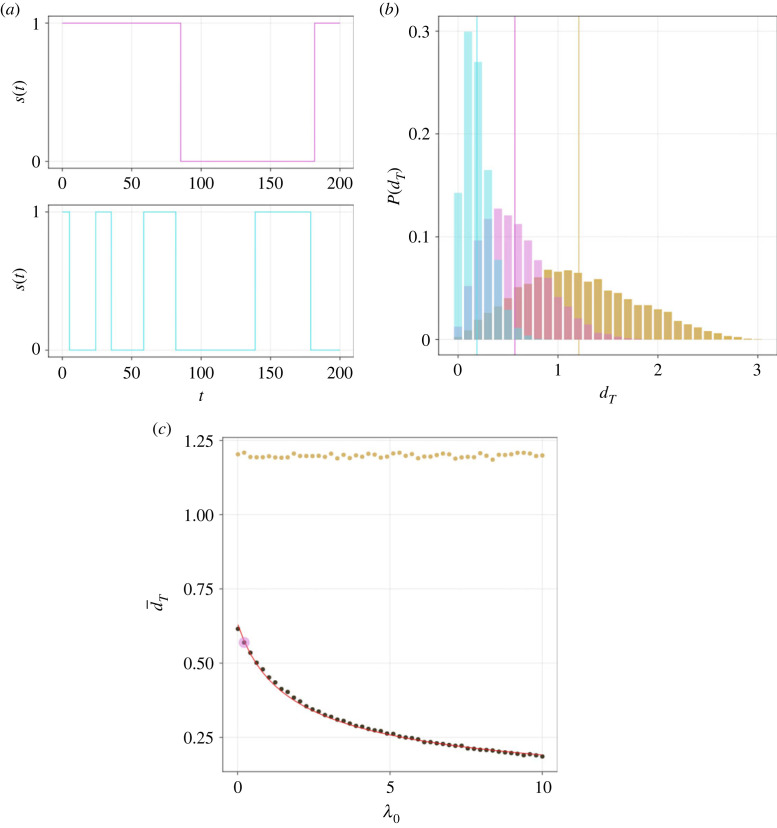
The effect of timer kinetics on spatial redistribution of the T6SS. (*a*) Realizations of the state variable, *s*(*t*), are plotted against time in the case of small (top, magenta) and large (bottom, cyan) *λ*_0_ (0.35 and 9.66, respectively). (*b*) Empirical PDFs, *P*, for MC displacement after time *T*, *d*_*T*_, are plotted for small (magenta) and large (cyan) *λ*_0_. Vertical lines denote estimated means. Yellow histogram denotes estimated PDFs in the case where MC positions are uniformly sampled from the spatial domain. (*c*) The mean displacement after time *T*, ${\bar{d}}_{T}$, is plotted against *λ*_0_ (red line denotes theoretical model; see equation (2.7)). Coloured markers denote small and large *λ*_0_. *T* = 10. Other parameter values as in table 2 unless otherwise stated.

To explore how the switching kinetics affect the redistribution of the T6SS, summary statistics that characterize T6SS displacement were computed. Reducing the parameter *λ*_0_ results in a T6SS spending relatively more time in State 0 (figure 4*a*). As T6SS motion arises in State 0, we expect that reducing *λ*_0_ will result in increased T6SS displacement. In figure 4*b*, we plot the measured T6SS displacements in a given simulation time, *T*, at two different values of *λ*_0_. Here it is clear that the simulations with larger *λ*_0_ have T6SSs that travel shorter distances. The displacement distribution is compared with a reference case in which the T6SS position at time *T* is uniformly sampled in the spatial domain. In figure 4*c*, the mean T6SS displacement is plotted against parameter *λ*_0_. A theoretical estimate for T6SS displacement (see equation (2.7)), derived in the case of an infinitely large domain, shows good agreement with the numerical computations. This arises for the chosen parameter values as the typical displacement of the T6SS in time *T* is smaller than the domain size.

**Figure 5.**
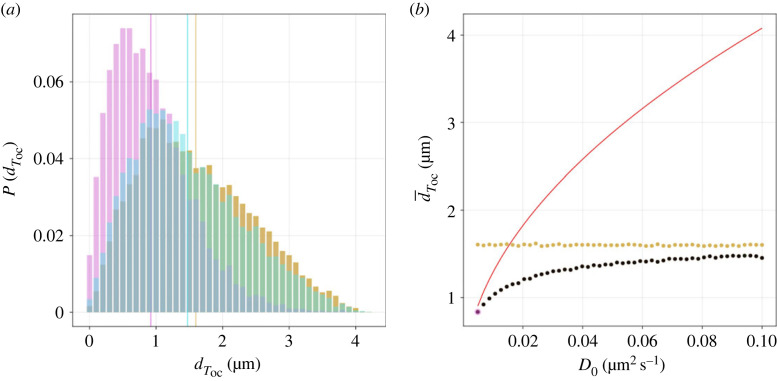
Spatial redistribution of the T6SS. (*a*) Empirical PDFs, *P*, for MC displacement after one firing cycle *T*_oc_, $d_{T_{\mathrm{oc}}}$, are plotted for small (magenta) and large (cyan) diffusion coefficient, *D*_0_ (0.0049 and 0.094 μm^2^ s^−1^, respectively). Vertical lines denote estimated means. Yellow histogram denotes estimated PDFs of the uniformly sampled domain. (*b*) The mean displacement, ${\bar{d}}_{T_{\mathrm{oc}}}$, is plotted against *D*_0_ (red line denotes theoretical model; see equation (2.7)). Coloured markers denote small and large *D*_0_. Other parameter values as in table 2.

To explore how changes to the T6SS hopping rate affect T6SS redistribution, simulations were performed with different values of the diffusion coefficient, *D*_0_. In each simulation run, the displacement of the T6SS was computed between successive fires of the T6SS (figure 5). When *D*_0_ is sufficiently large, the distribution of T6SS displacements tends to that of the uniformly sampled domain (i.e. a case where the hopping rate of the T6SS is so large that the location of the next firing event is uncorrelated with the previous firing event). The theoretical estimate for the mean displacement of the T6SS is only valid for sufficiently small values of the diffusion coefficient, *D*_0_ (figure 5*b*).

As definitive measurements of the T6SS firing position are not available in *S. marcescens*, a range of membrane diffusion coefficients that have been reported for different components of the membrane in a range of model microbial systems are considered (table 1). We label two limiting cases of simulated T6SS behaviour as ‘global’ and ‘local’ firing. Global firers (attained, for example, by making *D*_0_ sufficiently large) behave in a manner in which the locations of successive firing events are uncorrelated. By contrast, for a local firer, attained, for example, by making *λ*_0_ large, the position of the next firing event is strongly correlated with that of the previous firing event (figure 4*b*). It is found that the lower and upper ends of the diffusion coefficient range yield behaviour consistent with spatially local and global firing, respectively (figure 5*a*). In the case of large diffusion coefficients, it is observed that the distribution of T6SS displacement approaches that of the uniformly sampled model (figure 5*b*). These results suggest that the diffusion rate of the firing position is a parameter that could regulate the ability of T6SS to reorient between successive firing events.

## 4. Discussion

Secretion systems play a crucial role in the emergence of collective behaviours in bacterial communities. In the T6SS, a cell can dynamically assemble the T6SS machinery on a timescale of minutes. Upon firing, the attacker cell delivers its payload into a neighbouring cell, resulting in, among other responses, cellular damage or death. The kinetics of T6SS assembly and the ability of a T6SS to reorient are known to play an important role in the competitiveness of cells hosting the T6SS.

In this study, a computational model was developed that describes the kinetics of T6SS assembly as well as the ability of a T6SS to reorient between successive firing events. It was assumed that a T6SS can be in one of two states that represent distinct stages in its firing cycle. State 0 describes a motile MC that is not yet fully assembled into a T6SS. In State 1, the T6SS has fully formed, the position of the MC is fixed and the T6SS is in the process of firing. The firing of the T6SS is represented by a transition from State 1 to State 0. The model allows for exploration of how the kinetics of T6SS firing is related to the ability of the T6SS to reorient itself between successive firing events.

Using numerical simulations, we demonstrated that the distance travelled between successive fires is dependent on both kinetics of the T6SS assembly and the hopping rate (membrane diffusion coefficient). As relatively more time is spent in State 0, the average distance travelled between successive fires increases. The simulation results demonstrate that for sufficiently large hopping rates the T6SS behaves like a global firer: the position of the next firing event is largely uncorrelated with the position of the previous firing event. By contrast, for sufficiently small hopping rates the T6SS fires locally, i.e. in the vicinity of the previous firing event.

The model was parametrized using available data from *S. marcesens*. Whilst the T6SS kinetics have been well described, it is less clear what the membrane diffusion coefficient is in State 0. As a result, model behaviour was considered for a range of measured membrane diffusion coefficients. On the lower end of the range, the model behaves like a ‘local firer’: the distance travelled in State 0 is relatively small. By contrast, at the larger end of the range, the distance travelled in State 0 is relatively large and the system behaves like a ‘global firer’. These results suggest that the diffusion coefficient of the T6SS can play an important role in determining its spatial redistribution.

While it has been established that T6SS kinetics in *S. marcesens* are post-translationally regulated, here we consider a simplified model using a Markov jump process. Further work is required to describe the molecular regulation of T6SS. Specifically, a detailed exploration into the enzyme kinetics of the TPP-TagF regulation system may shed insight into the firing cycle mechanisms. Moreover, this study considers a model of T6SS assembly in an individual cell. To understand the role of spatio-temporal dynamics in a multi-cellular context (e.g. to apply the model to competition assays), the model needs to be extended to account for a more realistic description of cellular geometry and cell–cell interactions.

We have assumed that a Markov jump process describes the transition between states. As a result, the waiting times in each state are exponentially distributed. This allows for T6SS to spend arbitrarily small times in each of the states. Experimental measurement of waiting time distributions would allow this assumption to be validated/refined.

In future work, it would be interesting to investigate whether T6SS firing kinetics are cell density dependent. In the model, a single parameter change could enable switching between local and global firing modes in a manner that optimizes the efficiency of the attacking cell finding a target. Moreover, it would be interesting to investigate if the model can be generalized to account for tit-for-tat or duelling behaviours observed in other bacterial species. It could be speculated that these behaviours require localized firing, for which it could also be speculated that the diffusion of the MC or firing kinetics could be purposefully regulated.

Here we have formulated a simple spatio-temporal model of T6SS firing. The key insight from the model is that as a result of coupling between T6SS machine reorientation and the timer that regulates firing, the redistribution kernel is a function of both timer kinetics and membrane hopping.

## Data Availability

Code was developed using Julia [29]. Plots were generated using the package Makie.jl [30]. Data and relevant code for this research work are stored in GitHub: https://github.com/fieldofnodes/T6ssSpaceTimeRedistribution and have been archived within the Zenodo repository: https://zenodo.org/record/8356632 [31].
